# Ultrafast MRI and diffusion-weighted imaging: a review of morphological evaluation and image quality in breast MRI

**DOI:** 10.1007/s11604-025-01826-1

**Published:** 2025-07-04

**Authors:** Maya Honda, Masako Kataoka, Mami Iima, Marcel Dominik Nickel, Tsutomu Okada, Yuji Nakamoto

**Affiliations:** 1 Preemptive Medicine and Lifestyle-Related Disease Research Center, Kyoto University Hospital, 53 Kawahara-cho, Shogoin, Sakyo-ku, Kyoto, 606-8507 Japan; 2 Division of Surgery, Kansai Electric Power Medical Research Institute, Osaka, Japan; 3 Department of Fundamental Development for Advanced Low Invasive Diagnostic Imaging, Nagoya University Graduate School of Medicine, Nagoya, Japan; 4 Department of Diagnostic Imaging and Nuclear Medicine, Kyoto University Graduate School of Medicine, Kyoto, Japan; 5 Siemens Healthineers AG, Forchheim, Germany; 6 Department of Diagnostic Radiology, Kansai Electric Power Hospital, Osaka, Japan

**Keywords:** Breast neoplasms, Magnetic resonance imaging, Ultrafast, Diffusion-weighted imaging

## Abstract

Breast magnetic resonance imaging (MRI) is an essential tool for evaluating breast lesions, with dynamic contrast-enhanced (DCE) MRI being considered the reference standard. However, conventional DCE-MRI has limitations, including long scan times, high costs, and variable specificity leading to unnecessary biopsies. Emerging techniques such as ultrafast dynamic contrast-enhanced (UF-DCE) MRI and diffusion-weighted imaging (DWI) have recently received attention as possible alternatives. UF-DCE MRI achieves high temporal resolution, improving lesion conspicuity while reducing motion artifacts and background parenchymal enhancement. Advanced acceleration methods, including view sharing and compressed sensing, enhance temporal resolution while maintaining image quality. DWI, a contrast agent-free technique that can be used to assess tissue cellularity, provides high specificity in the differentiation of benign from malignant lesions. Recent developments in DWI, such as readout-segmented echo planar imaging, reduced field of view, and simultaneous multi-slice techniques, have significantly improved spatial resolution and reduced artifacts. These advancements enable morphological assessment and hold the potential for replacing or complementing conventional DCE-MRI, thus reducing patient burden and improving accessibility. Future research should focus on optimizing imaging protocols and integrating artificial intelligence to enhance diagnostic performance. This review discusses the principles, technological advancements, and clinical applications of UF-DCE MRI and DWI, with a particular focus on morphological evaluation and image quality, emphasizing their role in improving the efficiency of breast imaging while maintaining accuracy.

## Introduction

Breast magnetic resonance imaging (MRI) has become an indispensable tool for the diagnosis and evaluation of breast lesions. Conventional dynamic contrast-enhanced MRI (DCE-MRI) remains the reference standard for breast imaging, providing detailed morphological and kinetic data that adhere to Breast Imaging Reporting and Data System (BI-RADS) standards. However, limitations such as long acquisition times and high costs limit the accessibility to DCE-MRI. Relatively low specificity is another limitation of DCE-MRI that can lead to unnecessary biopsies [1].

Emerging techniques, including ultrafast dynamic contrast-enhanced (UF-DCE) MRI and diffusion-weighted imaging (DWI), aim to address these challenges. UF-DCE MRI improves temporal resolution, thereby facilitating dynamic imaging that captures the kinetic information of contrast inflow. Quantitative and semi-quantitative parameters obtained from UF-DCE MRI have been shown to have high diagnostic accuracy in discriminating malignant from benign breast lesions [2–4]. DWI can be used to evaluate tumor cellularity through water diffusion. The apparent diffusion coefficient (ADC) calculated from DWI yields high specificity in the distinction of breast cancer from benign breast lesions without the need to administer a contrast agent during MRI [5]. Both methods have the potential to complement conventional DCE MRI, and even to potentially replace it, thereby reducing patient burden. If morphological evaluation is also possible with these sequences alone, it may be possible to omit conventional sequences, shorten scan times, and improve patient comfort.

This review explores the principles, technical advancements, and clinical applications of ultrafast MRI and DWI in breast imaging, with a focus on their potential to supplant conventional methods while maintaining diagnostic accuracy and improving accessibility.

## Ultrafast MRI

### Principles and techniques

UF-DCE MRI is characterized by its ability to capture dynamic images with high temporal resolution, usually in less than 7 s for a whole imaging volume, which is about ten times faster than typical conventional DCE-MRI. Unlike conventional DCE-MRI, which relies on three-phase imaging involving pre-contrast, early post-contrast (< 2 min), and delayed post-contrast (> 5 min) acquisitions, UF-DCE MRI generally involves rapid acquisition during the inflow phase of contrast agents. Semi-quantitative parameters such as maximum slope (MS) and time to enhancement (TTE) are then extracted from the image time series and used to evaluate inflow kinetics. MS represents the %signal uptake/s at the steepest part of the time–intensity curve [6], whereas TTE represents the time between aortic and lesion enhancement [7]. A recent review article showed that temporal resolution shorter than 5 s improves the specificity of these semi-quantitative parameters for discriminating between malignant from benign breast lesions [4]. Fast scans acquired during the inflow phase in UF-DCE MRI can further reduce motion artifacts and parenchymal contrast-enhancement of normal breasts, which may further improve lesion conspicuity, especially in premenopausal and lactating patients [8–10]. Parallel imaging methods, such as generalized auto-calibrating partially parallel acquisition (GRAPPA) and sensitivity encoding (SENSE), can reduce scan times by utilizing multiple receiver coils to acquire the signal, but the temporal resolution is limited by the balance between acceleration factor and signal-to-noise ratio (SNR). More recently, techniques such as view sharing and compressed sensing (CS) have been used to achieve this goal.

### View-sharing technique

View sharing is a technique used to improve temporal resolution by sharing k-space data between consecutive image acquisitions. Based on the assumption that the peripheral region of k-space (which contains high spatial frequency information representing edges, details, and sharp transitions, with the center of k-space containing information on image contrast and brightness) can be shared, this method effectively reduces the sampling burden while enhancing temporal resolution by prioritizing the acquisition of central k-space data. View sharing improves temporal continuity without significantly compromising spatial resolution, making it particularly useful for dynamic imaging applications such as breast MRI. This technique category includes many common acquisition sequences, including differential subsampling with Cartesian ordering (DISCO), k-space weighted image contrast (KWIC), time-resolved angiography with interleaved stochastic trajectories (TWIST), time-resolved contrast kinetics imaging (TRICKS), time-resolved keyhole angiography (4D-TRAK), and most keyhole techniques.

DISCO combines variable density, pseudorandom k-space segmentation, and two-point Dixon fat–water separation for high spatiotemporal resolution breast DCE-MRI [11].

**Fig. 1:**
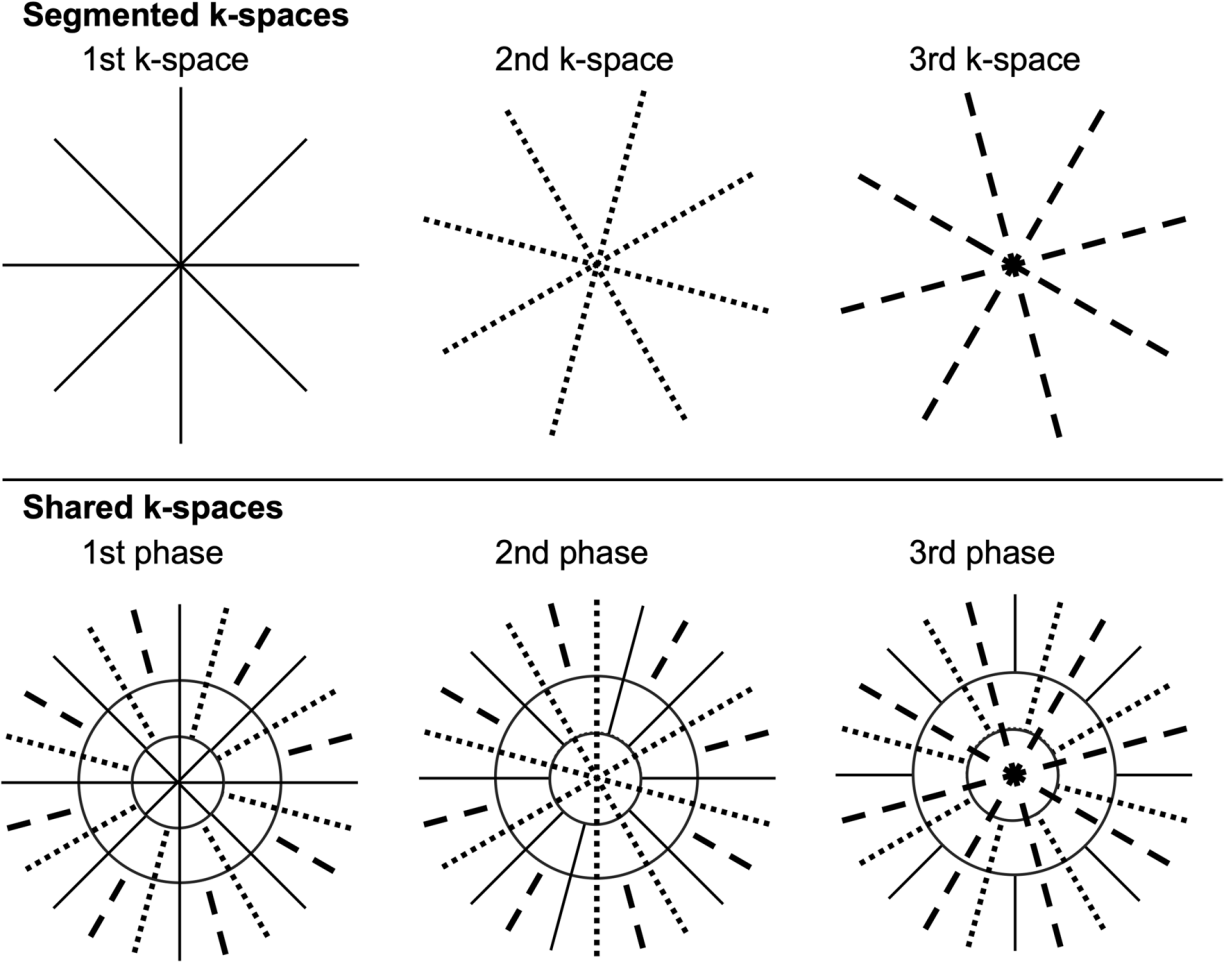
Example of k-space-weighted image contrast data acquisition (KWIC) in three-phase dynamic contrast-enhanced MRI with 12 spokes (four spokes/phase). The three k-spaces of each time phase share their periphery through the KWIC filter while primarily using data from closer time phases, balancing the sampling density between the center and the periphery (modified from [52])

KWIC leverages oversampling of the central k-space region in a radial acquisition, selectively integrating data from different temporal phases. During reconstruction, only the most recent acquisition is used for the central k-space, which dominates image contrast, while data from neighboring acquisitions progressively contribute to the outer k-space regions, preserving spatial detail while enhancing temporal resolution (Fig. 1). This weighted view-sharing approach effectively mitigates streaking artifacts and noise that arise from undersampled radial imaging, ensuring high-quality images with rapid temporal updates [12, 13].

**Fig. 2:**
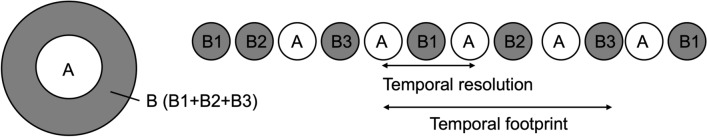
Example of time-resolved angiography with interleaved stochastic trajectories data acquisition in dynamic contrast-enhanced MRI with B% = 1/3. After acquiring the full k-space in the first measurement, a central portion A is acquired in each subsequent measurement together with a pseudo-determined portion in the periphery B. The missing portion of the B region is copied from the adjacent B acquisition for image reconstruction

**Fig. 3:**
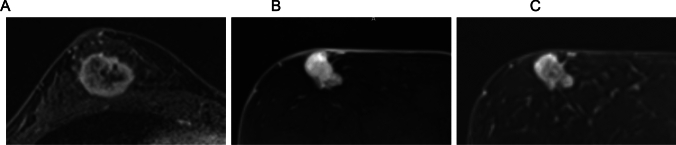
Time-resolved angiography with interleaved stochastic trajectories (TWIST) images with different ratios of central and peripheral regions. **A** TWIST-volumetric interpolated breath-hold examination (VIBE) with fat suppression (temporal resolution: 4.6 s, voxel size: 1 × 1 × 2.5 mm^3^, central region: 25%, peripheral region: 15%) of invasive ductal carcinoma exhibiting a mass lesion with rim enhancement. **B** TWIST-VIBE with fat suppression (temporal resolution: 4.6 s, voxel size: 0.7 × 0.7 × 2.5 mm^3^, central region: 30%, peripheral region: 33%) of invasive ductal carcinoma (different case from (A)). The internal enhancement pattern seems less heterogeneous compared with the VIBE reference image shown in (**C**) (voxel size: 0.9 × 0.9 × 1 mm^3^, 60–120 s from contrast injection, with fat suppression)

TWIST is an extension of TRICKS, and is a representative view-sharing technique that has been widely applied in breast UF-DCE MRI [2, 3]. It divides k-space into two regions: the central region A that contains low-frequency and high-contrast information, and the peripheral region B that holds high-frequency detail. During acquisition, the central k-space is fully sampled, enabling a shorter temporal resolution while maintaining adequate image contrast. Missing data in the peripheral k-space are copied from previous timepoints whose periphery is sampled using a different pseudo-stochastic trajectory (Fig. 2) [14]. The ratio of the central to peripheral region and the density parameter that further divides the B region, which can be chosen arbitrarily, affects the image quality; increasing the central region ratio improves image quality at the cost of temporal resolution, whereas larger number of subsampling of the periphery renders the method more prone to ringing artifacts (Fig. 3) 0.4D-TRAK is an application of the keyhole technique, where the k-space periphery is collected in the reference data set only at the end of the acquisition, and the resulting data is used for reconstruction of each dynamic phase [15].

### Compressed sensing 

**Fig. 4:**
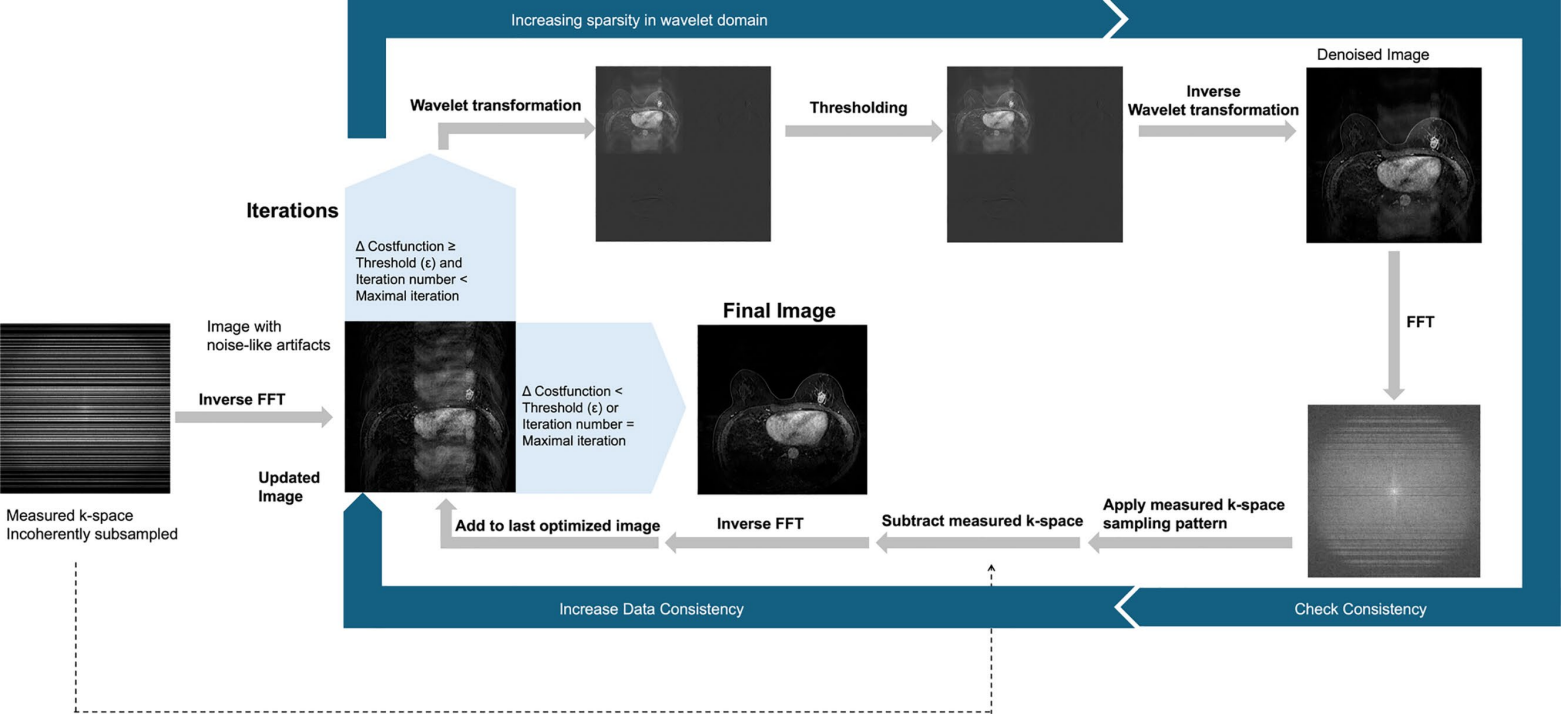
Schematic of image reconstruction using compressed sensing

CS is a rapid imaging technique that exploits the inherent sparsity of MR images in a transform domain to enable significant k-space undersampling while maintaining high image quality. CS reconstructs images from randomly undersampled k-space data by applying a nonlinear optimization process that enforces sparsity and data fidelity constraints. Unlike conventional undersampling, which introduces coherent aliasing artifacts, CS leverages incoherent sampling patterns that spread aliasing as noise-like interference, allowing iterative reconstruction algorithms to recover the image effectively (Fig. 4). By incorporating transform-domain sparsity constraints, CS enables accelerated MRI while preserving diagnostic accuracy, making it a valuable tool for high-resolution and time-resolved imaging applications [16].

**Fig. 5:**
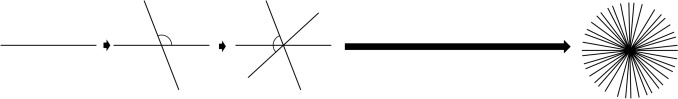
Radial k-space data collection for golden-angle radial sparse parallel MRI. Using the golden angle (111.25°) as the spoke angle increment for radial collection, the spokes do not overlap and always fill the least data-rich region of the kx-ky plane

**Fig. 6:**
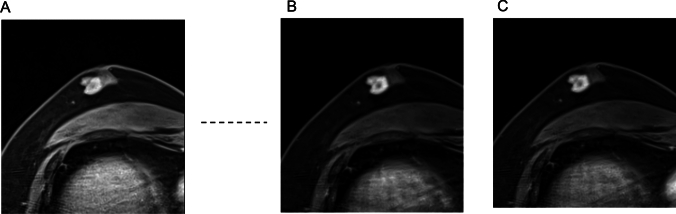
Golden-angle radial sparse parallel images with variable temporal resolution. Time resolution: **A** 44.8 s, **B** 6.5 s, **C** 4 s; spoke number: **A** 233, **B** 34, **C** 21; acceleration factor: **A** 3.5, **B** 23.7, **C** 38.3. The irregular shape and rim enhancement of the mass lesion is not much compromised by acceleration. Pathology results showed invasive ductal carcinoma of the male breast

Golden-angle radial sparse parallel MRI (GRASP) is a CS application that uses a radial stack-of-stars acquisition to achieve incoherent sampling [17]. Three-dimensional k-space data are collected in the kx-ky plane using a radial trajectory called a ‘spoke’, and in the kz direction using Cartesian phase encoding. The radial acquisition focuses data collection on the center of k-space, which affects image contrast and is less susceptible to motion artifacts. One of the features of GRASP is the use of the golden angle (111.25°) as the increment of the spoke angle. This ensures that the spokes do not overlap and always fill the region with the largest gap in the kx-ky plane (Fig. 5). Unlike data acquisition based on Cartesian sampling, this type of radial sampling allows for simultaneous uniform sampling of the angular distribution with different time resolutions. A feature of the GRASP-volumetric interpolated breath-hold examination (VIBE) is that any temporal resolution can be selected and images can be reconstructed at other temporal resolutions after imaging is complete (Fig. 6).

### Clinical applications including morphological evaluation

**Fig. 7:**
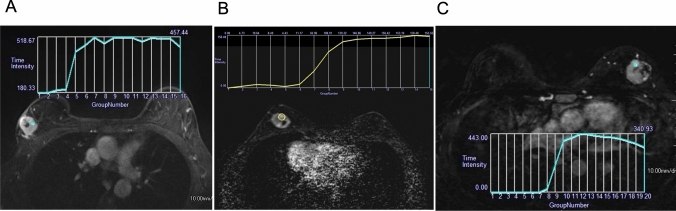
Comparison of time–intensity curves (TIC) across different ultrafast dynamic contrast-enhanced MRI techniques. The upslope of the TIC from the k-space-weighted image contrast (KWIC, time resolution: 3.8 s) is slightly jagged (**A**) compared to a time-resolved angiography with interleaved stochastic trajectories-volumetric interpolated breath-hold examination (VIBE) acquisition with a temporal resolution of 4.6 s (**B**), and compressed sensing-VIBE with a temporal resolution of 3.7 s (**C**). All mass lesions show rim enhancement and relatively steep upslopes indicative of malignancy. Pathology results showed invasive ductal carcinoma (**A** and **B**) and encapsulated papillary carcinoma (**C**), respectively

In the diagnosis of breast lesions, UF-DCE MRI has demonstrated comparable diagnostic accuracy to conventional DCE-MRI in terms of kinetic information such as the MS obtained from time–intensity curves [2, 4, 18]. Temporal blurring can cause blurring of time–intensity curves, which leads to false positives/negatives in the interpretation of kinetics, but smooth curves can be obtained from recent view-sharing sequences such as TWIST that share data from adjacent phases and CS. However, view-sharing sequences that share phases other than adjacent ones, such as KWIC, can yield jagged time–intensity curves due to temporal blurring (Fig. 7).

Although some studies have shown that kinetics from UF-DCE MRI have comparable diagnostic ability to morphology for distinguishing between breast cancer and benign breast lesions [19, 20], some types of malignant lesions such as invasive lobular carcinoma and ductal carcinoma in situ do not exhibit suspicious kinetics, and in these, morphology is still important for making the diagnosis, and it is, therefore, important to determine whether UF-DCE MRI can provide adequate morphological evaluation [2].

It is reported that higher spatial resolution can be obtained with CS than with view sharing [21]. A recent study used matched cohorts to compare two UF-DCE MRI sequences from two institutions; one was CS with a voxel size of 0.9 × 0.9 × 2.5 mm^3^ and a temporal resolution of 3.7 s, and the other was TWIST-VIBE with a voxel size of 0.7 × 0.7 × 2.5 mm^3^ and a temporal resolution of 8.5 s. Although aspects of the acquisition methods other than the acceleration also differed, such as contrast injection speed, the results suggested that CS may be superior to view sharing for morphological evaluation of breast lesions and depiction of blood vessels in the breast, whereas lesion conspicuity and time–intensity curve smoothness were comparable between the two acquisitions [22]. The results also showed that the presence or absence of fat suppression could affect certain artifacts; fat suppression may be defective when used, and motion artifact may be noticeable when subtraction images are used for evaluation without fat suppression.

**Fig. 8:**
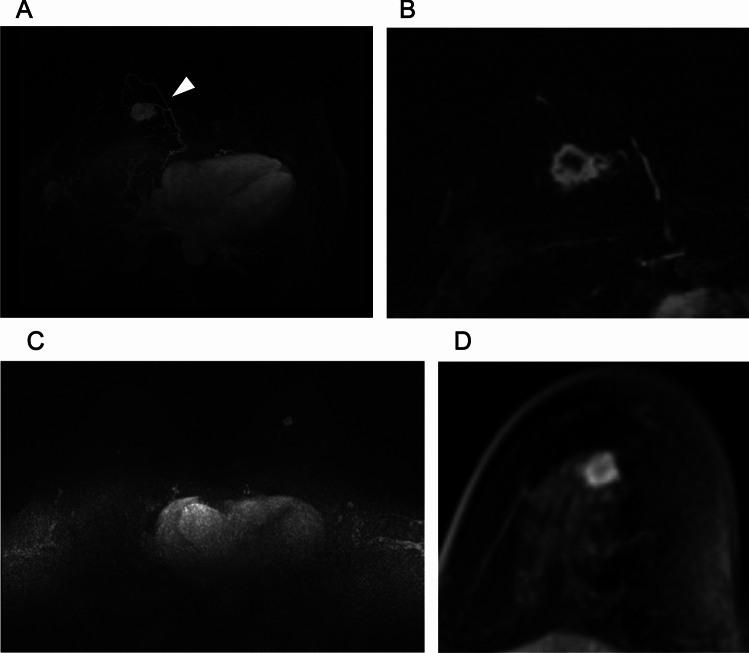
The maximum intensity projection and original image of a compressed sensing-volumetric interpolated breath-hold examination (VIBE) acquisition with temporal resolution: 3.7 s, voxel size: 0.9 × 0.9 × 2.5 mm^3^, last phase (**A** and **B**), and time-resolved angiography with interleaved stochastic trajectories-VIBE with temporal resolution: 8.5 s, voxel size: 0.7 × 0.7 × 2.5 mm.^3^, last phase (**C** and **D**) (reprint from [22])

Results have shown that clear delineation of tumor-associated blood vessels that may reflect the tumor microenvironment might be one feature of CS, and may lead to the diagnosis and classification of breast cancer [2]. Representative UF-DCE MRI images acquired using TWIST-VIBE and CS-VIBE are shown in Fig. 8.

**Fig. 9:**
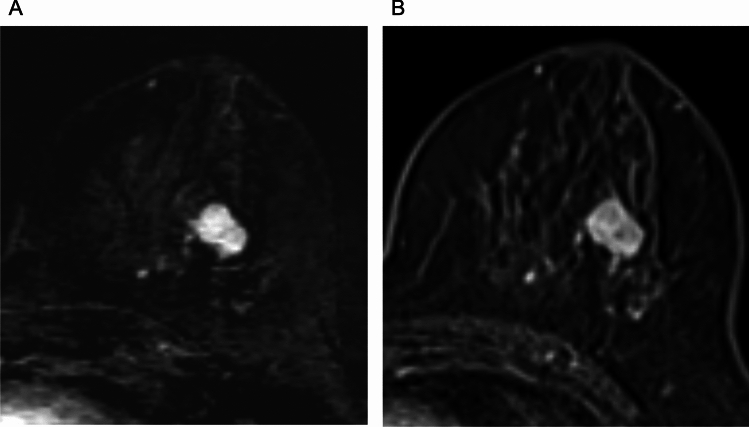
A case of intraductal papilloma with apocrine metaplasia. On compressed sensing-volumetric interpolated breath-hold examination (VIBE) (temporal resolution: 3.7 s, voxel size: 0.9 × 0.9 × 2.5 mm^3^, subtracting 1 st phase from the 20.^th^ phase) images, rim enhancement of the mass lesion is less obvious (**A**) compared to the early phase of three-dimensional VIBE with fat suppression (voxel size: 0.9 × 0.9 × 1 mm^3^) (**B**)

Ohashi et al. showed that UF-DCE MRI with CS-VIBE can potentially be used to evaluate morphological features of breast masses using the same criteria as conventional DCE-MRI, with excellent to almost perfect agreement in lesion size and shape [23]. However, discrepancies exist between the sequences, with UF DCE-MRI tending to describe mass lesions as having more circumscribed and less spiculated margins, and as being more homogeneous with less rim enhancement, all of which may suggest benignity (Fig. 9). Another study that included triple-negative breast cancer also showed that rim enhancement was less frequently observed with UF-DCE MRI than with conventional DCE-MRI [24]. In ductal carcinoma in situ, a clustered ring internal enhancement pattern is reported to be less frequently observed on UF-DCE MRI than on standard DCE-MRI [3].

**Fig. 10:**
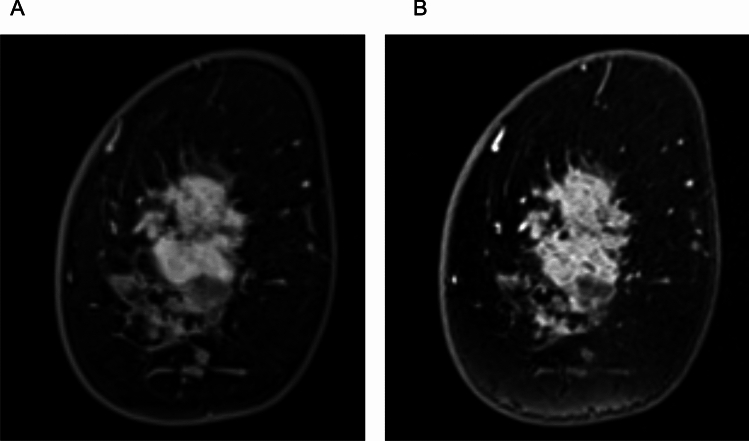
On golden-angle radial sparse parallel MRI with a voxel size of 0.8 × 0.8 × 0.9 mm^3^ and temporal resolution of 5.1 s (**A**), clustered ring internal enhancement of ductal carcinoma in situ is less obvious than on a volumetric interpolated breath-hold examination with a voxel size of 0.6 × 0.6 × 0.8 mm^3^ obtained at 2–5 min after contrast injection (**B**)

**Fig. 11:**
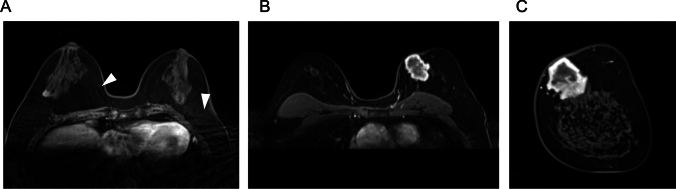
A case of invasive ductal carcinoma of the right breast presenting as a mass lesion (**A**). Streak artifacts are observed in the axial acquisition of golden-angle radial sparse parallel MRI (reconstructed voxel size: 0.7 × 0.7 × 2.5 mm^3^, acquired voxel size: 0.7 × 0.7 × 5.0 mm^3^ (**A**). A case of invasive ductal carcinoma of the left breast presenting as a mass lesion (**B**, **C**). Streak artifacts are less obvious in the axial multi-planar reconstruction from a coronal acquisition (reconstructed voxel size: 0.8 × 0.9 × 2.5 mm^3^, acquired voxel size: 0.8 × 1.4 × 2.5 mm^3^) (**B**) from coronal acquisition (reconstructed voxel size: 0.8 × 0.8 × 0.9 mm^3^, acquired voxel size: 0.8 × 0.8 × 1.4 mm^3^) (**C**)

GRASP is characterized by its high spatial and temporal resolution coming from CS and its optimal temporal resolution resulting from radial acquisition with the golden angle. Literature on the use of GRASP for morphological evaluation of breast lesions is limited, but GRASP is similar to CS-VIBE and the clustered ring may be ambiguous (Fig. 10). The radial acquisition used in GRASP generally results in streak artifacts, which are radially distributed lines, but GRASP reduces these streak artifacts through the use of compressed sensing [17]. In breast imaging, streak artifacts are seen centered on the axilla and heart, and sometimes extend a little into the breast, but they rarely affect the diagnosis of lesions. A coronal acquisition may be an option for GRASP if further artifact reduction is desired; because the streak artifacts extend in the acquisition plane, a coronal acquisition may reduce their effect in the breast (Fig. 11).

Representative UF-DCE MRI sequences are listed in Table 1.

**Table 1:** UF-DCE MRI study protocol in Japanese institutions (reprint from [3])

Institution*	Vendor	Scanner name	Magnetic field	Breast coil	UF protocol name**	Technique***	TR(ms)	TE(ms)	Flip angle(ﾟ)	Fat suppression	FOV (mm)	Orientation	Slice thickness (mm)
Kyoto Univ	Siemens	Prisma/Skyra	3 T	18-ch	Improved VIBE	CS	4.8	2.5	15	No	360 × 360	Axial	2.5
Saga Univ	Siemens	Prisma	3 T	18-ch	VIBE	PI	5.87	2.87	10	Yes	360 × 360	Axial	2.5
Saga Univ (2)	Siemens	Prisma	3 T	18-ch	Improved VIBE	CS	3	1.2	12	Yes	360 × 360	Axial	2.5
Nagoya Univ	Siemens	Prisma	3 T	18-ch	VIBE	CS	3.5	1.44	10	Yes	200 × 350	Axial	2
Dokkyo Univ	Siemens	Skyra	3 T	16-ch	TWIST	VS	6.17	2.94	10	Yes	330 × 330	Axial	2.0
KPUM	Siemens	Skyra	3 T	16-ch	TWIST-VIBE	VS	5.6	1.4	10	Yes	360 × 360	Axial	2.5
Tohoku Univ	Philips	Intera Achieva	3 T	16-ch	3D-FS-T1WI GRE	PI	2.8	1.5	10	Yes	350 × 350	Axial	4
TMDU	GE	Signa Pioneer	3 T	16-ch	DISCO	VS	3.7	1.3	10	Yes	360 × 360	Axial	4

Slice no	Voxel size (mm^3^)	Scan timing (injection start = 0 s)	Temporal resolution (sec)	Number of phases	Number of Iterations (in case of CS)	CS acceleration	Other parameters	Contrast agents****	Injection speed (ml/sec) *****
60	0.9 × 0.9 × 2.5	−13 to 60 s	3.7	20	30 times	16.5		Gadobutrol	2
48	0.9 × 0.9 × 2.5	0 to 99.6	8.3	12			GRAPPA factor 8	Gadobutrol	2.5
48	0.9 × 0.9 × 2.5	−8.41 to 89.9	2.9	31	30 times			Gadobutrol	2.5
72	0.78 × 0.78 × 2.0	−8.05 to 98.36 s	6.02	16	30 times	13.5		Gadobutrol	1
80	0.9 × 0.9 × 2.0	0 to 70 s	5	15			Gd-DOTA	Gadobenate dimeglumine	3
60	0.9 × 0.9 × 2.5	22 s (end of first frame of the TWIST)	5.3	17			GRAPPA factor 3	Gadoterate meglumine	2
80	1.09 × 1.66 × 4.0	10–64 s	3	18			SENSE acceleration factor 3.2(RL) 2.2 (FH)	Gadobenate dimeglumine	2
76	1 × 1.1	0 to 90 s	5.2	17				Gadobutrol	1

^*^KPUM Kyoto Prefectural University of Medicine, *TMDU*: Tokyo Medical Dental University

^**^VIBE volumetric interpolated breath-hold examination (VIBE), *TWIST* time-resolved angiography with interleaved stochastic trajectories, *DISCO* differential subsampling with Cartesian ordering

^***^PI parallel imaging, *VS* view sharing, *CS* compressed sensing

^****^Gadobutrol 0.1 ml/kg, gadobenate dimeglumine 0.2 ml/kg

^*****^followed by 20-40 ml saline flush

### Future directions

Evidence on morphological evaluation using UF-DCE MRI is still scarce, especially for non-mass lesions. Mass lesions can be evaluated morphologically on UF-DCE MRI with some limitations. In the future, integration of deep learning-based reconstruction techniques may further improve morphological detail and lead to wider clinical adoption of UF-DCE MRI.

## Diffusion-weighted imaging

### Principles and techniques

DWI is a non-contrast imaging modality that leverages the random motion of water molecules to assess tissue microstructure. The ADC, which can be simply calculated, provides quantitative insights into tumor cellularity and is widely adopted for evaluating breast lesions. ADC values are affected by acquisition parameters and environment, and therefore the European Society of Breast Imaging (EUSOBI) currently recommends the use of fat-suppressed single-shot echo planar imaging (ss-EPI) to evaluate ADC values in breast MRI [25]. However, ss-EPI is subject to specific artifacts and low spatial resolution, especially in the breast, where a large field of view (FOV) with a large amount of fat and air-tissue interfaces is required. Techniques such as parallel imaging, reduced FOV, and multi-shot EPI can help overcome these challenges.

Reduced FOV imaging uses a two-dimensional spatially selective radiofrequency excitation pulse and a 180° refocusing pulse to restrict the imaging area in the phase-encode direction. This method reduces the number of k-space lines needed, enabling higher in-plane resolution and minimizing off-resonance artifacts [26].

Multi-shot EPI (MS-EPI) DWI is a technique where k-space data are acquired in multiple excitations and can effectively reduce distortion and blurring along with a reconstruction method called image reconstruction using Image-Space Sampling function (IRIS) that can minimize motion effects [27, 28].

Readout-segmented EPI (rs-EPI) is a multi-shot EPI technique that is available commercially in the form of a product named readout segmentation of long variable echo-trains (RESOLVE). In RESOLVE, k-space is segmented into multiple shots along the readout direction, thereby reducing the effective echo spacing and mitigating distortions caused by field inhomogeneities. A key feature of RESOLVE is its use of two-dimensional navigator-based phase correction to compensate for motion-induced phase variations, thus ensuring artifact-free image reconstruction [29].

Multiplexed sensitivity encoding (MUSE) DWI is another multi-shot technique designed to mitigate motion-induced phase errors and improve image quality compared to conventional ss-EPI DWI [30]. MUSE integrates SENSE parallel imaging with a robust reconstruction algorithm that corrects for random phase variations between shots, thereby reducing geometric distortions and ghosting artifacts. By splitting the acquisition into multiple shots, MUSE achieves higher spatial resolution and improved SNR while maintaining clinically feasible scan times. Unlike navigator-based corrections, MUSE does not require additional acquisition steps, making it a practical option for high-resolution diffusion imaging.

Shot locally low-rank (shot-LLR) is also a multi-shot DWI reconstruction technique designed to mitigate phase variations between shots without explicit phase estimation. Unlike conventional methods that rely on navigator echoes or sensitivity encoding for phase correction, shot-LLR uses locally low-rank constraints to jointly reconstruct all shots while preserving high spatial resolution. This approach effectively reduces ghosting artifacts and improves image quality, particularly in high-resolution DWI acquisitions. Compared to MUSE, shot-LLR avoids explicit phase estimation by leveraging the redundancy in multi-shot k-space data. While MUSE is computationally more efficient and widely implemented, it is more susceptible to ghosting artifacts, especially with a higher number of shots. By contrast, shot-LLR provides superior artifact suppression and robustness against motion-induced phase variations, making it a promising technique for clinical breast MRI applications, albeit at the cost of increased computational complexity [31].

Simultaneous multi-slice (SMS) refers to excitation of multiple imaging slices at the same time. It typically uses a modulated radiofrequency pulse and makes use of the coil sensitivity in the through-slice direction to increase the number of acquired slices [32].

Spatiotemporal encoding (SPEN) is an advanced single-shot MRI technique that provides high-resolution imaging with enhanced robustness against distortion artifacts and susceptibility artifacts. Unlike conventional k-space encoding methods such as EPI, SPEN uses frequency-swept radiofrequency pulses combined with strong imaging gradients to achieve spatial encoding. This approach enables full refocusing of signals, thereby reducing T2* effects and mitigating geometric distortions. SPEN has demonstrated superior performance in breast DWI, achieving sub-millimeter resolution while maintaining accurate ADC measurements. By incorporating multiband pulses, parallel imaging, and high-bandwidth acquisition strategies, SPEN enables rapid high-fidelity imaging of malignancies with improved lesion delineation, particularly in challenging areas affected by fat or tissue interfaces [33, 34].

### Clinical applications including morphological evaluation

**Fig. 12:**
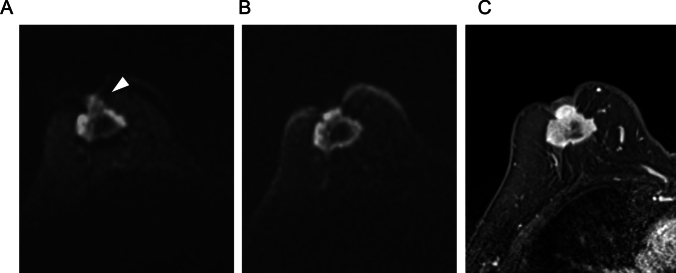
A case of invasive ductal carcinoma of the right breast just below the nipple presenting as a mass lesion. Distortion artifact below the nipple is more noticeable on single-shot echo planar imaging (ss-EPI) DWI with anterior to posterior phase-encoding direction (**A**) compared with single ss-EPI DWI with posterior to anterior phase-encoding direction (**B**). Compared to the early phase of three-dimensional volumetric interpolated breath-hold examination with fat suppression (voxel size: 0.9 × 0.9 × 1 mm^3^) (**C**), the tumor appears to have a similar morphology in (**B**), but in (**A**) the tumor appears to be extended toward the nipple

In the acquisition of ss-EPI DWI, care must be taken in respect to the phase-encoding direction, as distortion artifacts on DWI predominantly emerge in the phase-encoding direction because of its inherently lower bandwidth than the frequency-encoding direction (Fig. 12). Rodríguez-Soto et al. compared distortion artifacts in breast DWI acquired with different phase-encoding directions and polarities in both a phantom and patients [35]. Their results showed that radiologists preferred the posterior–anterior phase-encoding direction, even though this direction showed the highest median displacement in the phantom study.

Among advanced DWI techniques, Singer et al. compared reduced field of view (rFOV) and ss-EPI using a 1.5 T scanner and demonstrated that higher image quality and depiction of invasive breast cancer can be achieved using rFOV with an in-plane resolution of 1.1 mm and acquisition time of 4 min, with the compared ss-EPI having an in-plane resolution of 3.1 mm and acquisition time of 4.4 min [26]. MS-DWI using 3 T MRI provides robust high-resolution breast DWI with reduced geometric distortion compared to conventional SS-EPI DWI [36]. In bladder cancer imaging, it is reported to show less anatomical distortion than even rFOV [27].

**Fig. 13:**
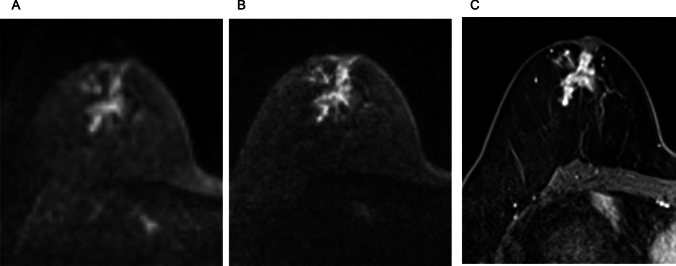
A case of invasive ductal carcinoma presenting as non-mass enhancement below the nipple. Compared to single-shot echo planar imaging (EPI) DWI (b = 1000 s/mm^2^ voxel size: 2 × 2 × 3 mm^3^, number of excitations (NEX): 2, 1.5 min/bilateral) (**A**), readout-segmented EPI DWI (b = 1000 s/mm^2^, voxel size: 1.3 × 1.3 × 3 mm.^3^, NEX: 1, 4 min/bilateral) has less distortion and more distinct morphology (**B**). **C:** Three-dimensional volumetric interpolated breath-hold examination for reference (voxel size: 0.9 × 0.9 × 1 mm, 60–120 s after contrast injection)

Using a 3 T scanner, Bogner et al. showed that RESOLVE with an in-plane resolution of 2.1 mm and acquisition time of about 3 min yielded higher image quality and lesion conspicuity than ss-EPI with the same resolution and acquisition time, achieving this higher quality by reducing distortion, blurring, and artifacts [37]. Other studies have also demonstrated the superior image quality of rs-EPI over ss-EPI in breast DWI using 3 T or 7 T scanners [38–40]. Kishimoto et al. compared tumor morphology on HR-RESOLVE (voxel size: 1.1 × 1.1 × 1.5 mm^3^; acquisition time: 5 min 15 s) with that on high-resolution contrast-enhanced (HR-CE) MRI (voxel size: 0.6 × 0.6 × 0.8 mm^3^), and reported a high agreement in the shape and margin of masses, as well as the distribution of non-mass enhancements (NMEs) [41]. The authors also found strong agreement in lesion size between HR-RESOLVE and HR-CE, as well as between HR-RESOLVE and pathological analysis [42]. However, rim enhancement of masses and clustered ring enhancement of NMEs, which were clearly visible on HR-CE, were often difficult to detect on HR-RESOLVE. Nevertheless, BI-RADS-like evaluation was reported to be feasible using HR-RESOLVE morphology and signal characteristics in combination with T1- and T2-weighted images, suggesting the potential for non-contrast MRI alone in breast lesion assessment [43]. Representative ss-EPI, RESOLVE, and CE-MRI images are shown in Fig. 13.

**Fig. 14:**
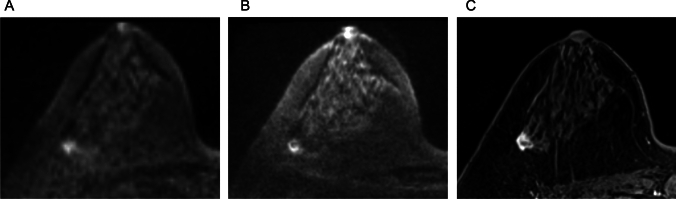
A case of invasive ductal carcinoma presenting a mass lesion. Compared to single-shot echo planar imaging (EPI) DWI (b = 1000 s/mm^2^, voxel size: 2 × 2 × 3 mm^3^, NEX: 2, 1.5 min/bilateral (**A**), intratumoral heterogeneity is depicted in simultaneous multi-slice readout-segmented EPI (b = 1000 s/mm^2^, voxel size: 1.3 × 1.3 × 3 mm^3^, NEX: 2, 4 min/bilateral) (**B**). **C:** Three-dimensional volumetric interpolated breath-hold examination with fat suppression for reference (voxel size: 0.9 × 0.9 × 1 mm^3^, 60–120 s after contrast injection)

Combining RESOLVE with SMS accelerates the acquisition while maintaining comparable image quality, SNR, and contrast-to-noise ratio (CNR) to conventional rs-EPI. In a study by Shin et al., SMS-RESOLVE demonstrated a 42% reduction in scan time (from 5:47 min to 3:20 min) compared with standard rs-EPI, with no significant loss in image clarity in breast MRI [44]. In the same study, radiologists rated SMS-RESOLVE as equal or superior to rs-EPI in over 70% of cases, with improvements in the distinction of anatomical structure and background noise reduction. ADC values were significantly higher on SMS-RESOLVE, potentially because of improved fat suppression and reduced partial volume effects [44]. These findings suggest that SMS-RESOLVE is a promising alternative to rs-EPI for high-resolution time-efficient DWI of the breast, with higher image quality than ss-EPI, although not directly compared. This supports the potential role of SMS-RESOVE in non-contrast MRI-based lesion assessment (Fig. 14).

Naranjo et al. compared MUSE DWI (in-plane resolution: 0.7 × 0.6 mm, acquisition time: 6 min) with conventional ss-EPI DWI (in-plane resolution: 1.3 × 1.3 mm, acquisition time: 4 min) in both phantom and patient imaging, and showed that MUSE DWI significantly improves image quality by reducing geometric distortions and enhancing SNR and CNR. In a cohort of 30 women with 37 breast lesions, MUSE DWI provided superior lesion visibility and better fat suppression while maintaining comparable ADC values to ss-EPI DWI [45]. Another study showed that MUSE and shot-LLR (in-plane resolution: 1 mm, acquisition time: 1 min 16 s for 4-shot, 2 min 22 s for 8-shot) demonstrated superior performance compared with single-shot DWI (in-plane resolution: 2.1 mm, acquisition time: 2 min 18 s), showing a significant improvement in SNR (*P* < 0.005), reduced distortion (*P* < 0.05), and enhanced apparent spatial resolution (*P* < 0.001). In addition, shot-LLR exhibited fewer ghost artifacts than both MUSE and ss-EPI DWI (*P* < 0.001). The reconstruction time for shot-LLR was about 1 min for each slice [31]. More recently, MUSE combined with deep learning reconstruction has been introduced, which yields a substantially better SNR than conventionally reconstructed MUSE, with no significant difference in CNR and ADC [46].

**Fig. 15:**
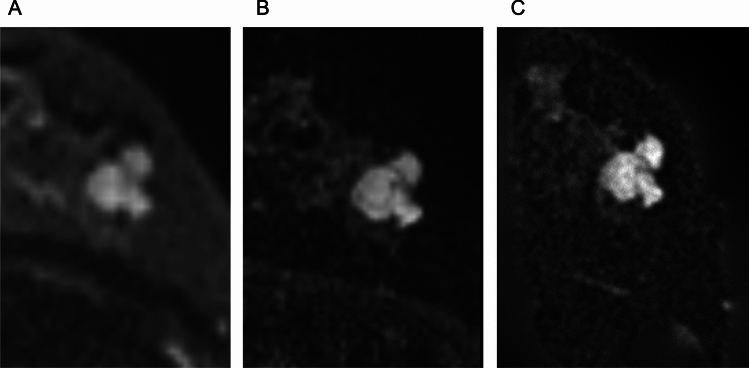
A case of benign phyllodes tumor presenting a lobulated mass lesion. The morphology of the cancer is clearly delineated on spatiotemporal encoding (b = 850 s/mm^2^, voxel size: 1 × 1 × 1.5 mm^3^, 2.3 min/three slices) (**C**) and readout-segmented echo planar imaging (EPI) (b = 850 s/mm^2^ 1.1 × 1.1 × 1.5 mm^3^, 5.3 min/unilateral images (**B**); conversely, it is relatively obscure on a single-shot EPI image (b = 1000 s/mm^2^, voxel size: 2 × 2 × 3 mm^3^, 1.1 min/bilateral) (**A**) (reprint from [47])

Iima et al. compared the conspicuity and ADC of breast lesions in ss-EPI, RESOLVE, and SPEN [47]. In their results, RESOLVE provided the highest lesion conspicuity among the three sequences, and the ADC values in breast lesions did not differ significantly between sequences obtained with b-values of 850–1000 s/mm^2^. However, SPEN with higher b-values (0, 850, 1500 vs. 0, 850 s/mm^2^) yielded significantly lower ADC values in malignant lesions, suggesting the importance of b-value selection in ADC quantification. Figure 15 demonstrates representative RESOLVE, SPEN, and ss-EPI DW images.

Representative breast DWI protocols other than ss-EPI are listed in Table 2.

**Table 2:** Representative breast DWI protocols other than ss-EPI

Author	Vendor	Scanner	Magnetic field	Breast coil	Technique	Voxel size, mm^3^	Orientation	Coverage
Kishimoto et al, Iima et al	Siemens	MAGNETOM Prisma	3 T	18ch	rsEPI	1.1 × 1.1 × 1.5	Sagittal	Unilateral breast
Yamaguchi et al	Siemens	MAGNETOM Prisma	3 T	18ch	rsEPI	2.1 × 2.1 × 3	Axial	Bilateral breasts
Shin Ahn et al	Siemens	MAGNETOM Vida	3 T	18ch	rsEPI	0.7 × 0.7 × 3	Axial	Bilateral breasts
Hu et al	Siemens	MAGNETOM Skyra	3 T	16ch	rsEPI	1.5 × 1.5 × 5	Axial	Bilateral breasts
Bogner et al	Siemens	Tim Trio	3 T	4ch	rsEPI	2.1 × 2.1 × 5	Axial	Bilateral breasts
Bogner et al	Siemens	Magnetom	7 T	4ch	rsEPI	0.9 × 0.9 × 5	Axial	Bilateral breasts
Wisner et al	Siemens	MAGNETOM Verio	3 T	8/16ch	rsEPI	1.8 × 1.8 × 2.4	Axial	Bilateral breasts
Shin Ahn et al	Siemens	MAGNETOM Vida	3 T	18ch	rsEPI, SMS	0.7 × 0.7 × 3	Axial	Bilateral breasts
Xiao et al	GE	SIGNA Premier	3 T	8ch	MUSE	2.7 × 2.0 × 4.0	Axial	Bilateral breasts
Naranjo et al	GE	Discovery MR750	3 T	16ch	MUSE	0.7 × 0.6 × 3.9	Axial	Bilateral breasts
Hu et al	GE	MR750	3 T	16ch	MUSE/shot-LLR	1 × 1 × 5	Axial	Bilateral breasts
Singer et al	GE	Signa HDx	1.5 T	8ch	rFOV	1.1 × 1.1 × 4	Axial/sagittal	8 slices/16 slices
Rahbar et al	Philips	NA	3 T	16ch	MS-DWI	1.2 × 1.2 × 4	Axial	Bilateral breasts
Iima et al	Siemens	MAGNETOM Prisma	3 T	18ch	SPEN	1 × 1 × 1.5	Axial	3 slices

TR (ms)	TE (ms)	Fat suppression	*b*-value (s/mm^2^)	Number of excitation/averages	Acceleration	Acquisition time, m:s	References
8300	48	SPAIR	0, 850	2	Parallel imaging factor 2	5:15	[41, 42, 47]
8190	41	SPAIR	NA	NA	NA	3:49	[50]
8860	61/91	CHESS	0, 1000	1	GRAPPA 2	5:47	[44]
5000	96	NA	0, 50, 1000, 2000	1	NA	4:27	[51]
8000	59	Inversion recovery; gradient reversal	0, 850	NA	NA	2:56	[37]
5500, 5800	62, 79	Three different approaches combined	0, 850	NA	GRAPPA 2 and none	3:35–40	[38]
8000–12000	64	Spectrally selective fat suppression	0, 800	NA	GRAPPA 2	< 5	[39]
4500	61/101	CHESS	0, 1000	1	GRAPPA 2, SMS 2	3:20	[44]
3074	53.3	NA	0, 1000	2	Parallel imaging factor 1; Number of shots 4	1:38	[46]
13,318	68.2	NA	0, 800	4	Acceleration 2; Number of shots 2	6:04	[45]
NA	NA	NA	0,600	NA	Parallel imaging factor 1; Number of shots 4, 8	1:16, 2:22	[31]
4000/3000	64.8	2D spatially selective RF excitation pulse and a 180° refocusing pulse	0, 800	16	NA	4, 6:36	[26]
6925	58/120	SPAIR	0, 800	1	Number of shots 2	2:53	[36]
6000	89	SPAIR	0, 850, 1500	2	NA	2:18	[47]

*TR* repetition time, *TE* echo time, *rsEPI* readout-segmented echo planar imaging, *RESOLVE* readout segmentation of long variable echo-trains, *MUSE* multiplexed sensitivity encoding, *shot-LLR* shot locally low-rank, *rFOV* reduced field of view, *SPEN* spatiotemporal encoding, *SPAIR* spectral attenuated inversion recovery, *CHESS* chemical shift selective, *GRAPPA* generalized auto calibrating partially parallel acquisition, *SMS* simultaneous multi-slice, *MS* multi-shot

### Future directions

Advanced sequences can mitigate the challenges of DWI, such as limited spatial resolution and susceptibility to artifacts. The integration of artificial intelligence with DWI in image acquisition was shown to contribute to reduced imaging time and improved image quality [48, 49], which holds promise for future widespread clinical use of breast DWI.

## Conclusion

Ultrafast MRI and DWI represent significant advancements in breast imaging through improved image quality while reducing scan times, reducing patient burden, and enhancing diagnostic specificity. Compressed sensing methods provide good morphological detail, though may mask certain malignant features by showing smoother tumor margins. Advanced DWI sequences significantly reduce distortion artifacts and match well with conventional MRI for tumor assessment. While they show potential to replace conventional DCE-MRI in certain scenarios, challenges such as standardization, resolution optimization, and widespread accessibility remain. Future research should focus on integrating these techniques with artificial intelligence to enhance diagnostic accuracy and clinical utility. By addressing these challenges, ultrafast MRI and DWI could potentially change breast imaging practice, improving outcomes for patients worldwide.
